# Seasonal variation in hemodialysis initiation: A single-center retrospective analysis

**DOI:** 10.1371/journal.pone.0178967

**Published:** 2017-06-02

**Authors:** Yujiro Maeoka, Takayuki Naito, Taisuke Irifuku, Yuka Shimizu, Takahiko Ogawa, Takao Masaki

**Affiliations:** 1 Department of Nephrology, Hiroshima University Hospital, Hiroshima, Japan; 2 Nephrology and Dialysis Division, Hiroshima Prefectural Hospital, Hiroshima, Japan; Universidade Estadual Paulista Julio de Mesquita Filho, BRAZIL

## Abstract

The number of new dialysis patients has been increasing worldwide, particularly among elderly individuals. However, information on seasonal variation in hemodialysis initiation in recent decades is lacking, and the seasonal distribution of patients’ conditions immediately prior to starting dialysis remains unclear. Having this information could help in developing a modifiable approach to improving pre-dialysis care. We retrospectively investigated the records of 297 patients who initiated hemodialysis at Hiroshima Prefectural Hospital from January 1st, 2009 to December 31st, 2013. Seasonal differences were assessed by χ^2^ or Kruskal-Wallis tests. Multiple comparison analysis was performed with the Steel test. The overall number of patients starting dialysis was greatest in winter (n = 85, 28.6%), followed by spring (n = 74, 24.9%), summer (n = 70, 23.6%), and autumn (n = 68, 22.9%), though the differences were not significant. However, there was a significant winter peak in dialysis initiation among patients aged ≥65 years, but not in those aged <65 years. Fluid overload assessed by clinicians was the most common uremic symptom among all patients, but a winter peak was only detected in patients aged ≥65 years. The body weight gain ratio showed a similar trend to fluid overload assessed by clinicians. Pulmonary edema was most pronounced in winter among patients aged ≥65 years compared with other seasons. The incidences of infection were modestly increased in summer and winter, but not statistically significant. Cardiac complications were similar in all seasons. This study demonstrated the existence of seasonal variation in dialysis initiation, with a winter peak among patients aged ≥65 years. The winter increment in dialysis initiation was mainly attributable to increased fluid overload. These findings suggest that elderly individuals should be monitored particularly closely during the winter.

## Introduction

The number of new dialysis patients, particularly among the elderly, has been increasing globally [1, 2]. In Japan, the number of new dialysis patients increased 1.8-fold from 20,877 in 1991 to 37,946 in 2011 [3]. The mean age at dialysis initiation also increased from 55.3 years in 1991 to 66.6 years in 2011. The most common age for patients to start dialysis in 2011 was 75–79 years, accounting for 16% of new dialysis patients, with 63% of patients aged ≥65 years. These changes may influence the timing of initiation of maintenance dialysis, because elderly patients tend to have more comorbidities [4] and are less able to tolerate uremic symptoms compared with younger patients [5, 6].

Several previous studies have reported significant increases in sodium intake [7] and body-weight gain [8] in winter, while other studies have noted a winter peak in some respiratory tract infections [9–11] and many cardiovascular diseases among elderly patients [12–15]. These events may be associated with deteriorating renal function, which may lead to the need for dialysis in some patients. Although seasonal variation in dialysis initiation has been reported previously [16], that study analyzed data from a historical data base from 1971 to 1990, and did not included information on patients’ conditions immediately prior to starting dialysis. Thus although the above changes over recent decades may have influenced the timing of dialysis initiation or the conditions of patients just before dialysis, a recent seasonal variations in dialysis initiation remain poorly understood. Many clinicians treat pre-dialysis chronic kidney disease (CKD) patients according to the guidelines of KDIGO or the society of nephrology of their own country. The recommendations in these guidelines are generally based on the results of high-quality randomized controlled studies. These recommendations are quite useful in helping clinicians to provide optimal medical services. However, randomized controlled trials rarely consider seasonal variations in clinical outcomes, such as dialysis initiation. Many studies also exclude very elderly pre-dialysis CKD patients. It is important to ascertain what is happening in the real world and to assess current practice. These investigations may help us to develop a modifiable approach to improving pre-dialysis care. Thus, we retrospectively investigated the seasonal variation in dialysis initiation and patients’ conditions just before starting dialysis, by age-stratified analysis.

## Materials and methods

### Study setting

The present study was conducted at Hiroshima Prefectural Hospital, which is a 712-bed tertiary hospital in Hiroshima City, Japan. Winter was defined as the period from December to February, spring as March to May, summer as June to August, and autumn as September to November, according to the Japan Meteorological Agency [17]. We obtained records of air temperature from January 2009 to December 2013 in the South Ward of Hiroshima City from the same source [17].

### Ethics statement

This study complied with the standards of the Declaration of Helsinki and current ethical guidelines, and was approved by the Ethical Committee of Hiroshima Prefectural Hospital (H28-N024). The committee waived the need for consent because this was a retrospective study, exclusively based on data extracted from hospital medical records, and all data were analyzed anonymously.

### Patients

This study included all 297 patients with end-stage kidney disease (ESKD) who initiated hemodialysis at Hiroshima Prefectural Hospital from January 1st 2009 to December 31st 2013. Data were retrospectively reviewed from hospital medical records to identify seasonal variation in dialysis initiation.

### Data sources

Demographic data such as age, sex, primary cause of ESKD, past history, body mass index, blood pressure, laboratory data, medication, and nephrology referral were drawn from medical records. Blood pressure and laboratory data at dialysis initiation were used. Cardiovascular disease was defined as follows: a history of angina, myocardial infarction, stroke, heart failure, or peripheral artery disease. Additionally, early nephrology referral was defined as a first nephrology visit ≥3 months prior to initiation of dialysis, and late nephrology referral as a first nephrology visit <3 months prior to initiation of dialysis [18]. To assess patients’ conditions immediately prior to dialysis, we also recorded the main uremic symptoms, and body weight gain ratio, and cases of infections, cardiac complications, pulmonary edema, scheduled dialysis or emergency dialysis from hospital medical records. The main uremic symptoms at dialysis initiation were assessed by clinicians and classified as fluid overload, general malaise, nausea or appetite loss, hyperkalemia, other uremic symptom, and no symptom. Fluid overload was defined as uncontrolled systemic edema, pleural effusion, ascites, and pulmonary edema. We calculated the body weight gain ratio to assess the fluid status as: body weight at dialysis initiation—dry weight at discharge / dry weight at discharge. Infectious cases were defined as those in which a causal pathogen was found, who were also considered to be critically ill, and who required empirical antimicrobial or antiviral therapy. Cardiac complications were defined as coronary artery disease necessitating coronary angiography, percutaneous coronary intervention, or open-heart surgery, and valvular heart disease diagnosed by echocardiography. Pulmonary edema was confirmed by physical examination and chest X-ray or computed tomography without apparent cardiac complications. Emergency dialysis initiation was defined as the need for a temporary vascular catheter without elective permanent vascular access, or patients requiring critical care regardless of elective permanent vascular access.

### Statistical analysis

We expressed nonparametric data as medians and interquartile ranges, and categorical variables as percentages. To compare the four seasons, we used the Kruskal–Wallis test for continuous variables. When we detected a significant difference among the four seasons, we performed multiple comparison analysis with the Steel test, setting winter as a control. We used χ^2^ tests for categorical variables. We performed several analyses by dividing the study population into two groups as follows: Group A included patients aged <65 years and Group B included those ≥65 years. A two-sided *P* value < 0.05 was considered statistically significant. Data were analyzed using JMP software (version Pro 12).

## Results

### Clinical characteristics

The clinical characteristics of all 297 patients at dialysis initiation are summarized in Table 1. The median age was 69 years, 199 patients (67%) were male, and 137 patients (46%) had diabetic nephropathy. There were no significant differences in characteristics among the seasons, except in albumin, hemoglobin, and C-reactive protein.

**Table 1:** Clinical characteristics.

	Total	Spring	Summer	Autumn	Winter	*P* value
**Patients, n**	297	74	70	68	85	0.51
**Age, years**	69 (61, 77)	70 (61, 78)	67 (57, 75)	67 (59, 77)	72 (64, 77)	0.11
**Male, n (%)**	199 (67)	50 (68)	45 (64)	46 (68)	58 (68)	0.55
**Diabetic nephropathy, n (%)**	137 (46)	40 (54)	30 (43)	31 (46)	36 (42)	0.60
**HT, n (%)**	276 (93)	69 (93)	65 (93)	64 (94)	78 (92)	0.62
**Dyslipidemia, n (%)**	128 (43)	34 (46)	23 (33)	34 (50)	37 (44)	0.31
**CVD, n (%)** ^†^	111 (37)	25 (34)	27 (39)	25 (37)	34 (40)	0.58
**Never smoker, n (%)**	139 (47)	32 (43)	33 (47)	37 (54)	37 (44)	0.90
**Former smoker, n (%)**	120 (40)	31 (42)	29 (41)	23 (34)	37 (44)	0.34
**Current smoker, n (%)**	38 (13)	11 (15)	8 (11)	8 (12)	11 (13)	0.81
**BMI, kg/m**^**2**^	23 (22, 27)	24 (21, 27)	23 (21, 25)	23 (22, 29)	24 (22, 26)	0.32
**SBP, mmHg**	161 (143, 177)	160 (143, 177)	161 (141, 175)	168 (145, 184)	158 (141, 172)	0.11
**DBP, mmHg**	80 (72, 90)	79 (71, 90)	80 (70, 89)	84 (75, 95)	79 (69, 88)	0.06
**Cr, mg/dL**	7.5 (5.0, 9.2)	7.8 (4.9, 9.2)	8.0 (5.7, 9.6)	7.2 (5.0, 9.1)	7.2 (4.5, 9.1)	0.50
**eGFR, mL/min/1.73m**^**2**^	6.1 (4.8, 8.9)	5.9 (4.7, 8.6)	5.9 (4.5, 8.2)	6.4 (4.9, 8.9)	6.5 (4.9, 9.6)	0.28
**Alb, g/dL**	3.3 (2.8, 3.7)	3.2 (2.8, 3.4)	3.5 (2.8, 3.8)	3.6^a^ (3.1, 3.9)	3.2 (2.8, 3.5)	0.002
**Hb, g/dL**	8.9 (8.1, 9.9)	8.8 (8.4, 9.6)	8.9 (7.8, 9.9)	9.5^a^ (8.5, 10.5)	8.6 (7.9, 9.8)	0.02
**CRP, mg/dL**	0.3 (0.2, 17)	0.3 (0.2, 1.2)	0.2 (0.2, 1.9)	0.2 (0.2, 1.2)	0.6 (0.2, 2.5)	0.049
**NT-proBNP, pg/mL**	7558 (2769, 19078)	8338 (3344, 19221)	6872 (2233, 20846)	5687 (2929, 14821)	7372 (2779, 23957)	0.91
**CCB, n (%)**	242 (81)	59 (80)	58 (83)	55 (81)	70 (82)	0.55
**ACEI/ARB, n (%)**	147 (49)	40 (54)	39 (56)	30 (44)	38 (45)	0.64
**Diuretics, n (%)**	184 (62)	43 (58)	41 (59)	43 (63)	57 (67)	0.31
**ESA, n (%)**	174 (59)	39 (53)	40 (57)	45 (66)	50 (59)	0.62
**Early nephrology referral, n (%)**	185 (62)	44 (59)	39 (56)	49 (72)	53 (62)	0.49
**Late nephrology referral, n (%)**	112 (38)	30 (41)	31 (44)	19 (28)	32 (38)	0.27

Data are median (25 percentile, 75 percentile) or number (percentage). HT, hypertension; CVD, cardiovascular disease; BMI, body mass index; SBP, systolic blood pressure; DBP, diastolic blood pressure; Cr, creatinine; eGFR, estimated glomerular filtration rate; Alb, albumin; Hb, hemoglobin; CRP, C-reactive protein; CCB, calcium channel blocker; ACEI, angiotensin converting enzyme inhibitor; ARB, angiotensin II receptor blocker; ESA, erythropoietin-stimulating agent. *P* value from χ^2^ test or Kruskal-Wallis test.

^a^ indicates *P* value < 0.05 versus winter.

### Mean air temperature trends

**Fig 1:**
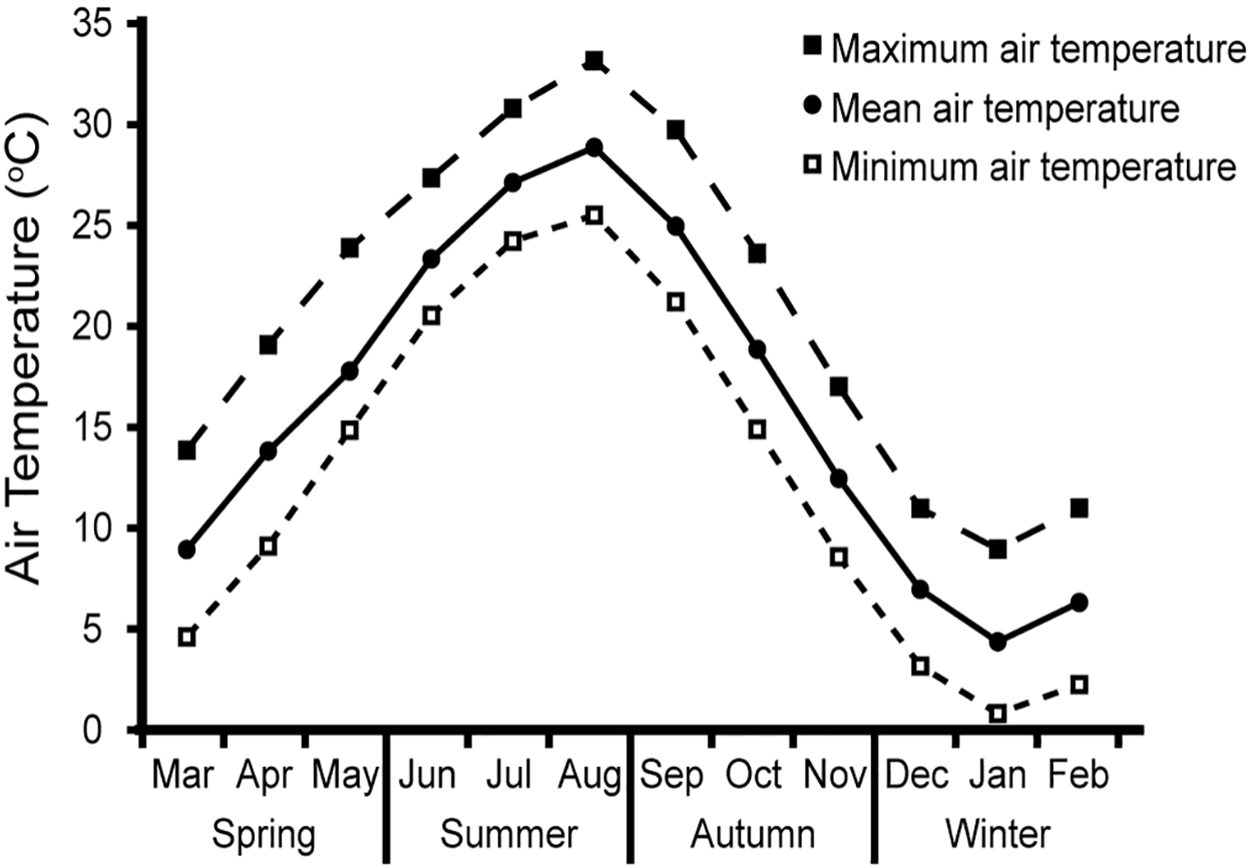
Monthly air temperature in Hiroshima, Japan from January 2009 to December 2013. Mean monthly air temperature (closed circles), maximum air temperature (closed squares), and minimum air temperature (open squares) in South Ward of Hiroshima City. Data obtained from the Japan Meteorological Agency.

The mean air temperature trends in Hiroshima City from 2009 to 2013 are shown in Fig 1. The mean air temperature was lowest in January and highest in August.

### Seasonal variations in dialysis initiation

**Fig 2:**
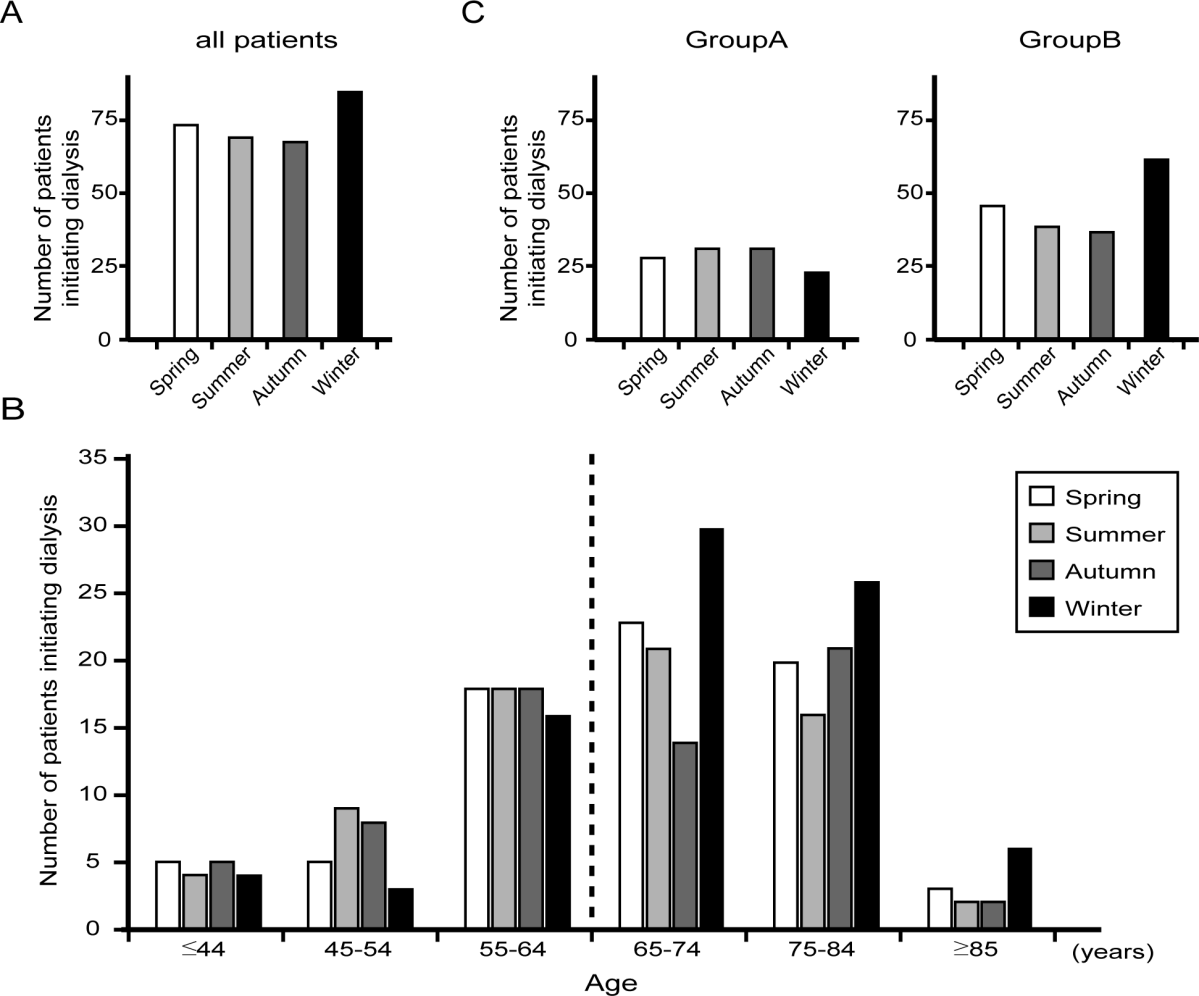
Seasonal variation in the number of patients initiating dialysis. (A) Number of all patients initiating dialysis. (B) Number initiating dialysis according to age. (C) Number initiating dialysis among patients aged <65 years (Group A) (left) and ≥65 years (Group B) (right).

Dialysis initiation was most frequent in winter, followed by spring, summer, and autumn (Table 1). Although there were no significant seasonal differences among all patients, there appeared to be a winter peak among patients aged ≥65 years (Fig 2A and 2B). We therefore divided the cohort into two groups: Group A included patients aged <65 years (n = 113, 38%) and Group B included those ≥65 years (n = 184, 62%). Age-stratified analysis revealed a significant winter peak of dialysis initiation among individuals in Group B but not Group A (Fig 2C).

### Seasonal variations in condition at dialysis initiation

**Fig 3:**
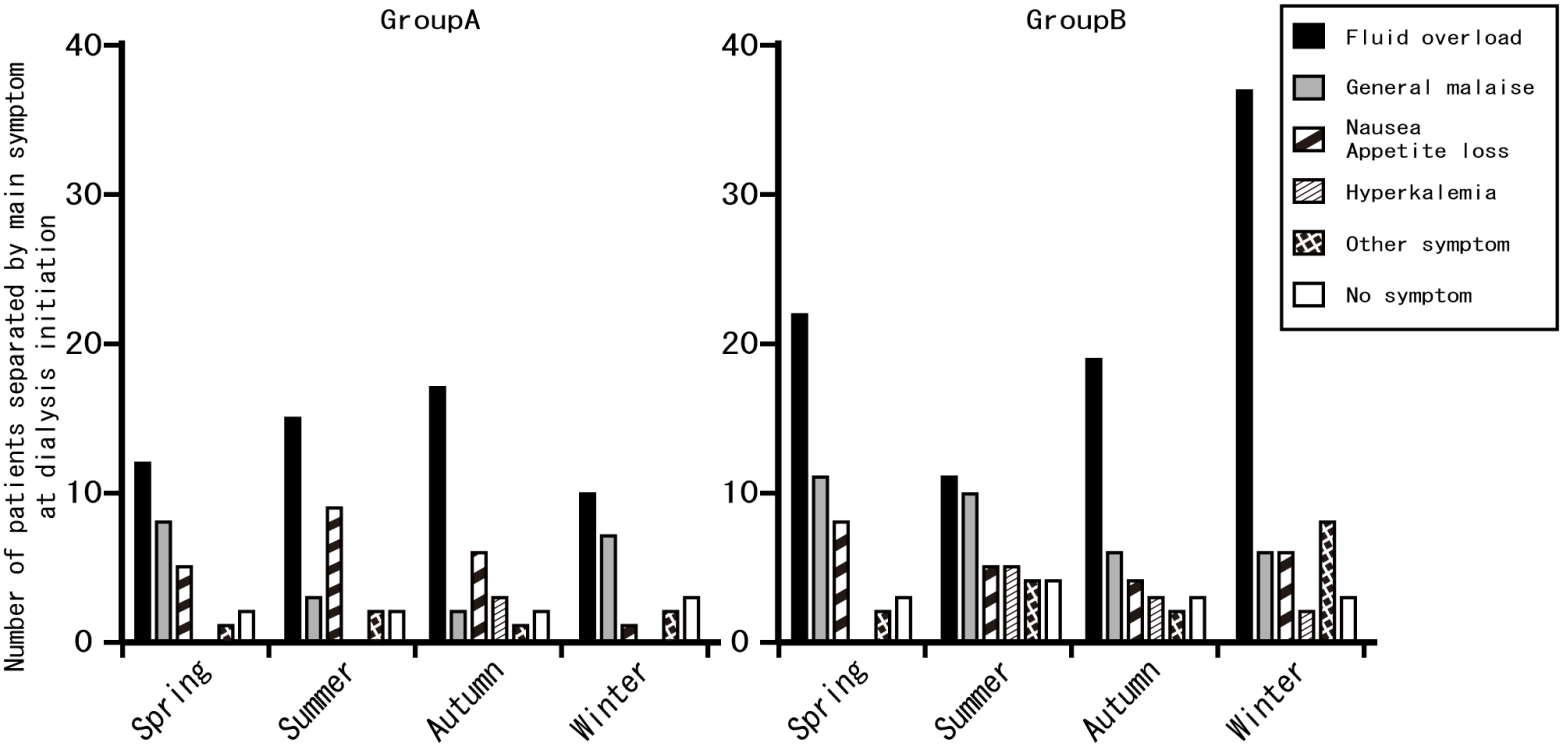
Seasonal variation in number of patients according to main symptoms at dialysis initiation. Number of patients with fluid overload, general malaise, nausea or appetite loss, hyperkalemia, other uremic symptoms, and no symptom at dialysis initiation among Groups A and B.

Although fluid overload assessed by clinicians was the most common symptom among all patients, a winter peak was only detected in Group B (Fig 3). The body weight gain ratio showed a similar trend to fluid overload assessed by clinicians (Table 2). Pulmonary edema was most pronounced in winter among Group B compared with other seasons, and although infections were slightly increased in winter among Group B, the difference was not significant. There were no seasonal variations in cardiac complications in either age group.

**Table 2:** Seasonal variations in conditions at dialysis initiation.

	Group A						Group B					
	Total	Spring	Summer	Autumn	Winter	*P* value	Total	Spring	Summer	Autumn	Winter	*P* value
**Patients, n**	113	28	31	31	23	0.68	184	46	39	37	62	0.04
**Age, years**	58 (51, 62)	58 (49, 62)	56 (52, 61)	58 (50, 62)	59 (49, 62)	0.88	75 (70, 79)	75 (70, 80)	73 (70, 78)	77 (73, 82)	75 (70, 78)	0.20
**Male, n (%)**	85 (75)	20 (71)	25 (81)	23 (74)	17 (74)	0.63	114 (62)	30 (65)	20 (52)	23 (62)	41 (66)	0.03
**Diabetic nephropathy, n (%)**	60 (53)	19 (68)	14 (45)	15 (48)	12 (52)	0.63	77 (42)	21 (46)	16 (41)	16 (43)	24 (39)	0.49
**HT, n (%)**	101 (89)	24 (86)	28 (90)	29 (94)	20 (87)	0.57	175 (95)	45 (98)	37 (95)	35 (95)	58 (94)	0.06
**Dyslipidemia, n (%)**	49 (43)	14 (50)	12 (39)	14 (45)	9 (39)	0.71	79 (43)	20 (43)	11 (28)	20 (54)	28 (45)	0.06
**CVD, n (%)** ^†^	37 (33)	9 (32)	10 (32)	9 (29)	9 (39)	0.99	74 (40)	16 (35)	17 (44)	16 (43)	25 (40)	0.38
**Never smoker, n (%)**	45 (40)	14 (50)	12 (39)	14 (45)	5 (22)	0.18	94 (51)	18 (39)	21 (54)	23 (62)	32 (52)	0.20
**Former smoker, n (%)**	42 (37)	7 (25)	12 (42)	11 (35)	11 (48)	0.70	78 (42)	24 (52)	16 (41)	12 (32)	26 (42)	0.08
**Current smoker, n (%)**	26 (23)	7 (25)	6 (19)	6 (19)	7 (30)	0.98	12 (7)	4 (9)	2 (5)	2 (5)	4 (6)	0.72
**BMI, kg/m**^**2**^	25 (22, 28)	24 (21, 26)	24 (22, 27)	27 (22, 32)	25 (20, 26)	0.87	23 (21, 26)	24 (22, 28)	22 (20, 24)	23 (21, 26)	24 (22, 26)	0.09
**SBP, mmHg**	163 (142, 181)	165 (144, 181)	166 (146, 182)	162 (143, 181)	151 (132, 169)	0.45	161 (143, 175)	156 (142, 175)	156 (140, 169)	171* (157, 189)	160 (143, 173)	0.03
**DBP, mmHg**	85 (76, 96)	84 (72, 95)	86 (79, 97)	89 (77, 98)	86 (73, 93)	0.63	78 (68, 86)	78 (69, 86)	75 (67, 85)	84 (73, 89)	76 (66, 86)	0.13
**Cr, mg/dL**	8.3 (6.0, 10.1)	8.3 (5.9, 10.1)	8.3 (6.4, 11.0)	7.9 (5.1, 9.2)	8.5 (6.3, 9.9)	0.87	6.8 (4.6, 8.8)	7.1 (4.7, 8.8)	6.8 (5.1, 9.2)	5.7 (4.6, 8.6)	6.7 (4.0, 8.8)	0.66
**eGFR, mL/min/1.73m**^**2**^	5.9 (4.7, 8.4)	5.2 (4.2, 8.7)	5.9 (4.4, 6.8)	6.2 (4.8, 9.3)	5.7 (4.9, 8.0)	0.87	6.4 (5.0, 9.1)	6.0 (5.1, 8.6)	5.7 (4.5, 8.3)	6.5 (5.4, 8.8)	7.2 (4.8, 10.5)	0.27
**Alb, g/dL**	3.2 (2.8, 3.7)	3.1 (2.7, 3.4)	3.3 (2.7, 3.8)	3.5 (2.9, 3.9)	3.2 (2.9, 3.6)	0.21	3.4 (2.8, 3.7)	3.3 (2.9, 3.5)	3.6^a^ (3.1, 3.8)	3.6^a^ (3.3, 3.9)	3.2 (2.8, 3.5)	0.002
**Hb, g/dL**	9.2 (8.4, 10.1)	9.0 (8.4, 9.5)	9.2 (8.3, 10.4)	9.4 (8.5, 10.6)	9.0 (8.1, 10.0)	0.68	8.8 (7.9, 9.8)	8.8 (8.3, 9.6)	8.4 (7.8, 9.9)	9.6^a^ (8.6, 10.4)	8.5 (7.8, 9.6)	0.015
**CRP, mg/dL**	0.2 (0.2, 1.2)	0.3 (0.2, 0.9)	0.2 (0.2, 0.9)	0.2 (0.2, 0.8)	0.8 (0.2, 2.2)	0.29	0.5 (0.2, 2.1)	0.3 (0.2, 1.3)	0.2 (0.2, 2.3)	0.4 (0.2, 1.4)	0.6 (0.2, 3.2)	0.28
**NT-proBNP, pg/mL**	6271 (2606, 20728)	5670 (384, 29710)	10093 (2099, 23185)	4698 (2965, 12730)	7396 (1837, 23866)	0.94	8037 (3123, 18961)	8358 (4836, 19110)	6467 (2144, 17156)	7559 (1264, 18980)	7347 (3158, 24320)	0.90
**CCB, n (%)**	89 (79)	22 (79)	26 (84)	26 (84)	15 (65)	0.30	153 (83)	37 (80)	32 (82)	29 (78)	55 (88)	0.01
**ACEI/ARB, n (%)**	55 (49)	16 (57)	16 (52)	14 (45)	9 (39)	0.50	92 (50)	24 (52)	23 (59)	16 (43)	29 (47)	0.29
**Diuretics, n (%)**	66 (58)	15 (54)	18 (58)	18 (58)	15 (65)	0.91	118 (64)	28 (61)	23 (59)	25 (68)	42 (68)	0.06
**ESA, n (%)**	63 (56)	13 (46)	18 (58)	19 (61)	13 (57)	0.58	111 (60)	26 (57)	22 (56)	26 (70)	37 (60)	0.21
**Early nephrology referral, n (%)**	72 (64)	17 (61)	17 (55)	22 (71)	16 (70)	0.75	113 (61)	27 (59)	22 (56)	27 (73)	37 (60)	0.24
**Late nephrology referral, n (%)**	41 (36)	11 (39)	14 (45)	9 (29)	7 (30)	0.46	71 (39)	19 (41)	17 (44)	10 (27)	25 (40)	0.09
**Fluid overload, n (%)**	54 (48)	12 (43)	15 (48)	17 (55)	10 (43)	0.54	89 (48)	22 (48)	11 (28)	19 (51)	37 (60)	0.001
**Body weight gain ratio, %**	7.3 (2.2, 14.3)	3.9 (0.7, 12.1)	7.5 (2.7, 14.9)	8.8 (3.4, 12.5)	6.4 (2.7, 14.5)	0.25	6.7 (2.2, 12.2)	6.8 (1.9, 13.8)	2.7^a^ (0.9, 10.6)	4.9 (2.3, 11.4)	8.0 (4.7, 13.9)	0.01
**Pulmonary edema, n (%)**	12 (11)	2 (7)	4 (13)	4 (13)	2 (9)	0.72	22 (12)	4 (9)	3 (8)	3 (8)	12 (19)	0.02
**Infection, n (%)**	19 (17)	5 (18)	6 (19)	3 (10)	5 (22)	0.80	35 (19)	6 (13)	10 (26)	5 (14)	14 (23)	0.12
**Cardiac complications, n (%)**	8 (7)	1 (4)	2 (6)	2 (6)	3 (13)	0.80	13 (7)	1 (2)	4 (10)	5 (14)	3 (5)	0.44
**Scheduled dialysis, n (%)**	65 (58)	18 (64)	15 (48)	17 (55)	15 (65)	0.94	104 (57)	28 (61)	22 (56)	26 (70)	28 (45)	0.82
**Emergent dialysis, n (%)**	48 (42)	10 (36)	16 (52)	14 (45)	8 (35)	0.34	80 (43)	18 (39)	17 (44)	11 (30)	34 (55)	0.002
**Use of temporary vascular catheter, n (%)**	48 (42)	10 (36)	16 (52)	14 (45)	8 (35)	0.34	72 (39)	18 (39)	16 (41)	8 (22)	30 (48)	0.003

Data are median (25 percentile, 75 percentile) or number (percentage). *P* value from χ^2^ test or Kruskal-Wallis test.

^a^ indicates *P* value < 0.05 versus winter.

We further investigated seasonal variations in scheduled and emergent dialysis initiation in each group, and detected a significant winter peak in emergent cases only among Group B (Table 2). Use of temporary vascular catheters showed a similar trend to emergent dialysis initiation.

## Discussion

The number of new dialysis patients has been increasing worldwide, particularly among elderly individuals [1, 2]. These changes may require additional improvements in pre-dialysis care. In this study, we found a winter peak in dialysis initiation among patients aged ≥65 years. A similar seasonal variation in dialysis initiation was observed in 1,982 patients with ESKD in Okinawa, Japan, between 1971 and 1990 [16], with a close association between the incidence of ESKD and ambient temperature. However, the mean age of that study population was 56.9 years, which was younger than our cohort, and the ambient temperature was warmer, because Okinawa is located on the southern edge of Japan. In addition, that study did not consider the patients’ conditions just before starting dialysis, and the reasons for the observed seasonal variation remained to be clarified. Furthermore, pre-dialysis treatment has become more sophisticated in recent decades, in line with the spread of knowledge about CKD. We therefore conducted this study, which revealed a winter peak in dialysis initiation associated with worsening fluid overload among patients aged ≥65 years. Our results may help to develop improvements in current pre-dialysis care. First, elderly CKD patients should consider more frequent weight checks and stricter sodium restriction in winter than in other seasons. Second, clinicians should consider planning rigorous and frequent medical checks for older patients in winter. Third, although we found no significant seasonal variation in late referral, the finding that approximately 40% of new dialysis patients received nephrology care only within the previous 3 months needs to be addressed. Earlier referral to a nephrologist and multidisciplinary management could help to maintain kidney function and avoid deterioration in patient condition. Fourth, these results may help nephrologists determine sufficient staff numbers and the need for additional emergent dialysis units in the winter.

We found a close association between a winter peak in dialysis initiation and fluid overload as assessed by clinicians. Although clinicians’ assessments are generally reliable, numerical data supporting the validity of these assessments are preferable. We calculated the body weight gain ratio of patients and found a seasonal variation that mirrored clinicians’ assessments. This trend was reported previously by Yanovski et al., who reported that healthy subjects gained body weight during the period from mid-November to mid-January [8]. Other studies have found a winter peak in inter-dialytic weight gain among hemodialysis patients [19, 20]. Although the reason some people gain weight in winter has not been fully elucidated, one possible mechanism is a seasonal change in daily sodium intake. Shahar et al. reported a significant increase in sodium intake in winter based on a semi-quantitative food questionnaire [7], and Hata et al. also reported that daily urinary sodium levels were significantly higher in winter among Japanese hypertensive patients [21]. Japanese people tend to overeat during the Christmas and New Year holidays. Furthermore, older Japanese people like to eat high-salt soups (e.g. nabe-ryori, oden, udon, soba) and salted vegetables in winter. Although we did not examine sodium-intake records among our cohort, these habits may contribute to excessive salt intake and lead to progressive systemic edema through increased extracellular volumes. Progressive systemic edema may be one cause of weight gain in the winter among elderly pre-dialysis CKD patients. Although we did not find significant differences in spring and autumn with non-parametrical multiple comparison tests, this result may be attributed to the small sample size.

We found a seasonal variation in albumin and hemoglobin concentrations in Group B; levels of both were low in winter. We speculate that this variation may be associated with the dilution effect of excessive extracellular volume. Unfortunately, we could not confirm data related to actual fluid balance in this study, because we did not perform bioimpedance analysis as a routine test at dialysis initiation. NT-proBNP is another measurable variable related to congestive conditions. However, we did not observe a seasonal variation in NT-proBNP levels. Levels of NT-proBNP are influenced by residual renal function and comorbid cardiovascular disease [22]. The median eGFR at dialysis initiation was approximately 6 mL/min/1.73 m^2^ and the prevalence of comorbid cardiovascular disease was nearly 40% in the present study. These conditions may have contributed to a substantial elevation in NT-proBNP levels. It may be difficult to use excessive NT-proBNP levels to assess volume disturbances.

Cold-induced vasoconstriction may also help to explain the winter increase in dialysis initiation. Fagius et al. reported that muscle nerve sympathetic activity and blood pressure were increased in cold compared with warm environments [23], and Izzo et al. showed that systemic vascular resistance and plasma norepinephrine were higher in winter compared with summer [24]. Furthermore, sympathetic nerve activities are known to accelerate with age [25] and CKD [26]. On the basis of this information, we speculate that cold weather can cause sympathetically mediated vasoconstriction, which may lead to decreased renal perfusion and increased sodium resorption via activation of the intrarenal renin-angiotensin II-aldosterone system. As a result, elevated systemic blood pressure may damage vulnerable residual glomeruli, contributing to the rapid deterioration of kidney function in patients with advanced CKD.

We also observed a significant increase in emergent dialysis as a result of pulmonary edema during the winter among older patients. Hirai et al. recently reported a significant increase in acute heart failure during the winter in patients with systolic blood pressure >140 mmHg, but not in those with systolic blood pressure ≤140 mmHg [27]. Another report showed little body-weight change during pulmonary edema in some patients, suggesting that pulmonary edema may occur as a result of fluid redistribution, with no outstanding systemic volume overload [28, 29]. We confirmed that 9/12 (75%) older patients with pulmonary edema had blood pressure > 140/90 mmHg at dialysis initiation, and 5/12 (42%) patients showed mild to moderate body-weight changes < 10% (data not shown). Age-related reduced capacitance in the venous system may be associated with volume redistribution, though it is difficult to measure venous capacitance in a clinical setting. Although most patients did not start dialysis because of pulmonary edema, we should be aware that fluid redistribution as a result of neurohormonal activation may be a factor in some older CKD patients.

In this study, the winter increment in dialysis initiation was not associated with infections; although respiratory infections such as pneumonia and influenza showed a slight winter peak among older patients, the difference was not significant. The median C-reactive protein level among all patients was modestly higher in winter than in the other seasons, however the elevation was not significant among elderly patients either according to the Kruskal–Wallis test or multiple comparison tests. This lack of significant elevation might be associated with the increasing use of influenza and pneumococcal vaccinations among elderly Japanese CKD patients, but we could not confirm the vaccination records in the current study cohort.

This study had several limitations. First, the sample size was relatively small. Second, it was a single-center study conducted at a tertiary hospital, and possible selection bias could therefore not be excluded. Third, the study design was cross-sectional and the data were collected retrospectively. Fourth, depressive symptoms and quality of life are worse in the winter among maintenance hemodialysis patients [30]. Depression can cause deterioration of general conditions among pre-dialysis patients. However, we could not obtain numerical data on the quality or prevalence of depression, because this study was performed retrospectively and there was insufficient information related to these issues. Further research is needed to clarify the relationship between depression and seasonal variations in dialysis initiation. Finally, the external validity of this study is limited. Our results may be applicable only in regions with air temperature trends similar to those shown in Fig 1.

## Conclusions

Seasonal variation in dialysis initiation may exist among older patients, associated with worsening fluid overload. These findings indicate the need for intensive monitoring of elderly CKD patients in the winter.
